# A new species of *Elephantomyia* crane fly (Diptera, Limoniidae) from Jeju Island, South Korea

**DOI:** 10.3897/zookeys.966.48590

**Published:** 2020-09-09

**Authors:** Sigitas Podenas, Virginija Podeniene, Tae-Woo Kim, A-Young Kim, Sun-Jae Park, Rasa Aukštikalnienė

**Affiliations:** 1 Nature Research Centre, Akademijos str. 2, LT-08412 Vilnius, Lithuania Life Sciences Centre of Vilnius University Vilnius Lithuania; 2 Life Sciences Centre of Vilnius University, Sauletekio str. 7, LT-10257 Vilnius, Lithuania Nature Research Centre Vilnius Lithuania; 3 Animal Resources Division, National Institute of Biological Resources, Incheon 22689, South Korea National Institute of Biological Resources Incheon South Korea

**Keywords:** Eastern Palaearctic, habitat, key, larva, Limoniinae, taxonomy

## Abstract

A new species of crane fly (Diptera, Limoniidae), Elephantomyia (Elephantomyia) hallasana Podenas & Podeniene, **sp. nov.**, from Jeju Island, South Korea is described. Adult and larval characters are illustrated. Elephantomyia (E.) hallasana**sp. nov.** is the only species of the genus *Elephantomyia* Osten Sacken, 1860 recorded from Jeju Island, South Korea. Habitat, elevation range, and seasonality data are presented. Distributional notes on *E. subterminalis* Alexander, 1954 in the Far East of Russia (Khabarovskiy and Primorskiy regions) are discussed. An identification key for all Eastern Palaearctic species of subgenus E. (Elephantomyia) is presented.

## Introduction

Crane flies belonging to the genus *Elephantomyia* Osten Sacken, 1860 are easily recognized by their long proboscis, which often exceeds body length (head + thorax + abdomen). The extended rostrum, with relatively small mouth parts and reduced palpi, is used for sucking nectar from tubular flowers (Savchenko 1986). Previously, only one species of the genus *Elephantomyia* was recorded from the Korean peninsula (Podenas et al. 2015). Crane fly specimens collected during field trips to South Korea in 2017 and 2019 included one new species of *Elephantomyia*. It is the only species of the genus *Elephantomyia* recorded from Jeju Island.

## Materials and methods

All specimens of Korean crane flies of the genus *Elephantomyia* presented in this study are preserved at the National Institute of Biological Resources (**NIBR**), Incheon, South Korea; all other specimens mentioned are preserved at the National Museum of Natural History, Smithsonian Institution, Washington, DC, USA (**USNM**). Adults were collected by insect net and with collecting lights. Collected specimens were dry mounted laterally on paper points. Wet specimens are preserved in 96% ethanol (EtOH). Male wings were slide mounted in Euparal. Dissected male genitalia were cleared in 10% KOH and preserved in microvials with glycerol beneath the pinned specimen. Larvae were collected by hand digging in dead wood and preserved in 70% ethanol. Larval head capsules and spiracular fields were slide mounted in glycerol.

Specimens were examined with an Olympus SZX10 dissecting microscope and Nikon Eclipse T*i* microscope. Photographs of adults and larvae of Korean crane flies were taken with a Canon EOS 80D digital camera through a Canon MP-E 65 mm macro lens at the Nature Research Centre, Vilnius, Lithuania. Photographs of larval head capsules were taken with a Nikon DS-Fi1 digital camera at Vilnius University, Lithuania. Photographs for Figures 21–26 were taken with a Canon EOS 6D digital camera through a Canon MP-E 65 mm macro lens at the Academy of Natural Sciences of Drexel University, Philadelphia, PA, USA.

Terminology for adult morphological features follows McAlpine (1981). Larva morphological features follows Oosterbroek and Theowald (1991) and Teskey (1981). Definitions of biogeographical units follows Oosterbroek (2020).

## Taxonomy

### Elephantomyia

Taxon classificationAnimaliaDipteraLimoniidae

Osten Sacken, 1860

C25A3586-7D94-52A5-ABF2-A1D8F23AC7D9

Elephantomyia Osten Sacken, 1860: 220; Alexander 1948: 522; Ito 1948: 89; Ishida 1959: 2; Savchenko and Krivolutskaya 1976: 74; Savchenko 1983: 62, 1986: 202, 1989: 49; Podenas et al. 2015: 70.

#### Type species.

*Limnobiorhynchus canadensis* Westwood, 1836 (= *westwoodi* Osten Sacken, 1869).

A total of 16 species of *Elephantomyia* are known from the East Palaearctic (Oosterbroek 2020). They belong to two subgenera: E. (Elephantomyia) Osten Sacken, 1860 and E. (Elephantomyodes) Alexander, 1923. The nominate subgenus includes 14 species, and the subgenus Elephantomyodes includes two species (*E. sophiarum* Ito, 1948 from Honshu and Kyushu, Japan, and *E. tianmushana*Zhang et al., 2015 from Zhejiang, China). Eastern Palaearctic species of the subgenus E. (Elephantomyodes) can be easily distinguished from species belonging to the subgenus E. (Elephantomyia), as they have snowy white tarsal segments and a very narrow anal angle of the wing. Only one species of the genus *Elephantomyia*, *E. edwardsi* Lackschewitz, 1932 was previously recorded from the Korean Peninsula (Podenas et al. 2015).

### Key to the Eastern Palaearctic species of the subgenus Elephantomyia (Elephantomyia)

**Table:** 

1	General body color black	**2**
–	General body color yellow, brown (Fig. 1), or gray; but if thorax black, abdomen yellow	**4**
2	Wing clear, except stigma	**3**
–	Wing with distinct darkening surrounding cross-veins, along frontal margin, and along vein *Cu* (Fig. 21)	**Elephantomyia (Elephantomyia) carbo carbo Alexander, 1938a (China: Sichuan)**
3	Abdomen black	**Elephantomyia (Elephantomyia) insolita Alexander, 1940 (China: Sichuan)**
–	Abdomen bicolored: basal half of each segment yellow, distal dark brown	**Elephantomyia (Elephantomyia) palmata Alexander, 1947 (Japan: Honshu)**
4	Thorax dark (black, gray, or brown) (Fig. 1)	**5**
–	Thorax light (yellow or brownish yellow)	**10**
5	Rostrum as long as the remainder (head + thorax + abdomen) of the body	**6**
–	Rostrum shorter, approximately as long as abdomen (Fig. 1)	**8**
6	Antenna black	**Elephantomyia (Elephantomyia) inulta Alexander, 1938b (China: Xizang, Yunnan; India: Assam)**
–	Antenna yellow to brownish yellow (Fig. 1)	**7**
7	Tarsal segments yellow. Outer gonostylus of male terminalia with hooked apex, anterior apodeme of aedeagus large, fan-shaped	**Elephantomyia (Elephantomyia) laohegouensis Zhang, Li & Yang, 2015 (China: Sichuan)**
–	Tarsal segments brown. Outer gonostylus of male terminalia with bifid apex, anterior apodeme of aedeagus very small, tripartite	**Elephantomyia (Elephantomyia) zonata Savchenko, 1976 (Russia: Sakhalin, Kuril Island)**
8	Thorax gray. Aedeagus of male terminalia shaped as a long, coiled tube, gonostyli terminal	**Elephantomyia (Elephantomyia) plumbea Alexander, 1954 (Japan: Shikoku)**
–	Thorax brown (Fig. 1). Aedeagus of male terminalia short and nearly straight, if it is shaped as a long, coiled tube, then gonostyli situated medially on gonocoxite (Fig. 2)	**9**
9	Male gonostyli at the apex of gonocoxite, aedeagus short, not coiled (Fig. 23)	**Elephantomyia (Elephantomyia) tetracantha Alexander, 1954 (Russia: Primoskyi Kray; Japan: Shikoku)**
–	Male gonostyli situated medially on gonocoxite (Fig. 2), aedeagus long, coiled	**Elephantomyia (Elephantomyia) hallasana Podenas & Podeniene, sp. nov. (Korea: Jeju Island)**
10	Wing with costal area distinctly darkened (Fig. 24)	**Elephantomyia (Elephantomyia) hokkaidensis Alexander, 1924 (Russia: Amur Oblast, Primorskiy Kray, Sakhalin, Kuril Island; Japan: Hokkaido, Honshu, Shikoku)**
–	Wing with costal area not darker than entire wing (Fig. 7)	**11**
11	Antennal flagellum brown (Japanese species only)	**12**
–	Antennal flagellum yellow	**13**
12	Mesonotal prescutum with median anterior darkening, rostrum as long as abdomen, knob of halter pale yellow, paramere of male terminalia with few spines, mesal surface of gonocoxite covered with long sparse setae (Fig. 26)	**Elephantomyia (Elephantomyia) dietziana Alexander, 1930 (Japan: Honshu, Shikoku, Kyushu)**
–	Mesonotal prescutum without markings, rostrum as long as the remainder (head + thorax + abdomen) of the body, knob of halter darkened, paramere of male terminalia spineless, mesal surface of gonocoxite covered with short dense setae	**Elephantomyia (Elephantomyia) takachihoi Ito, 1948 (Japan: Kyushu)**
13	Apex of male gonocoxite with triangle shaped lobe beyond base of outer gonostylus, aedeagus long, coiled, paramere widened distally, distal part with straight spines (Figs 3, 4)	**14**
–	Apex of male gonocoxite widely rounded, not extended into lobe, aedeagus short and straight, not coiled, paramere long and narrow, without spines at apex	**Elephantomyia (Elephantomyia) krivosheinae Savchenko, 1976 (Russia: Altay, Tuva, Khabarovskiy kray, Primorskiy kray, European part; Europe)**
14	Paramere of male terminalia with 4 or 5 spines distally (Fig. 4)	**Elephantomyia (Elephantomyia) subterminalis Alexander, 1954 (Russia: Khabarovskiy kray, Primorskiy kray; Japan: Shikoku)**
–	Paramere of male terminalia with 12–16 spines distally (Fig. 3)	**Elephantomyia (Elephantomyia) edwardsi Lackschewitz, 1932 (Korea; Russia: Altay, European part; Europe)**

### Elephantomyia (Elephantomyia) hallasana

Taxon classificationAnimaliaDipteraLimoniidae

Podenas & Podeniene sp. nov.

76A618C2-F2C1-5255-9F78-8903D7F17031

http://zoobank.org/F9D288A8-FFD6-4504-AC1C-8A7A37A47C3A

Figs 1 , 2 , 6 , 8–19

#### Type species.

***Holotype***: Male (pinned), South Korea, Jeju-do, Seogwipo-si, Saekdal-dong, 33°21.46'N, 126°27.85'E, alt. 1100 m, 2019.06.17, S. Podenas leg., (NIBR).

**Figures 1–7.**
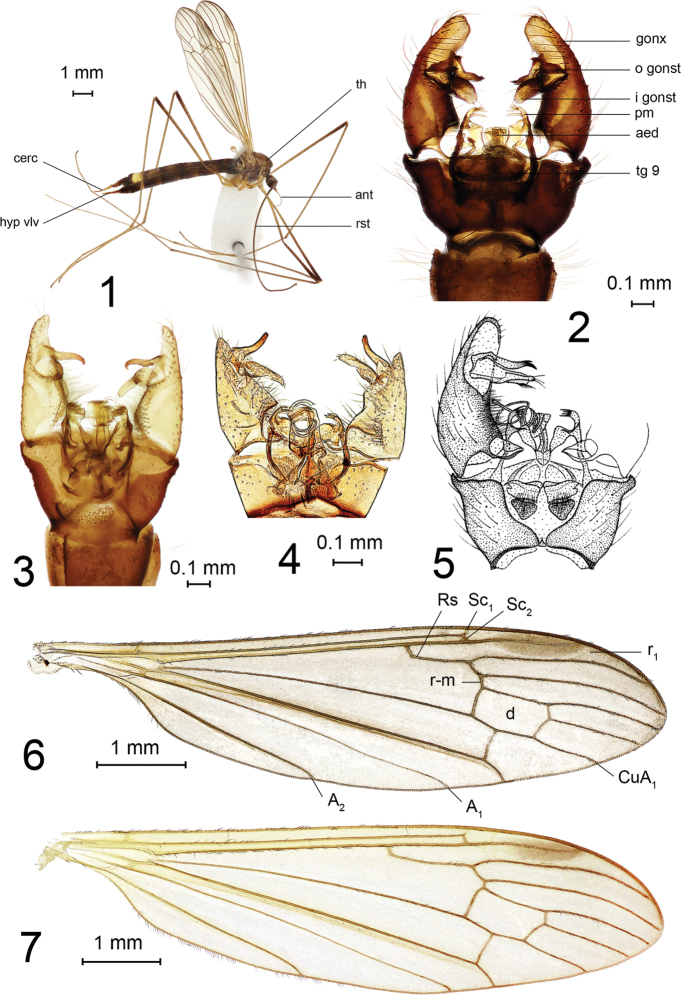
Elephantomyia (Elephantomyia). **1**E. (E.) hallasana Podenas, Podeniene, sp. nov., paratype, female, habitus view **2**E. (E.) hallasana sp. nov., paratype, male genitalia, dorsal view **3**E. (E.) edwardsi, male genitalia, dorsal view **4**E. (E.) subterminalis, holotype, male genitalia, dorsal view **5**E. (E.) sp., male genitalia, dorsal view (identified as *E. subterminalis* in Savchenko, 1986: fig. 101) **6**E. (E.) hallasana sp. nov., paratype, wing **7**E. (E.) edwardsi, wing. Abbreviations: A_1_ – first anal vein, A_2_ – second anal vein, aed – aedeagus, ant – antenna, cerc – cercus, CuA_1_ – branch of cubital anal vein, d – discal cell, gonx – gonocoxite, hyp vlv – hypogynial valva, i gonst – inner gonostylus, o gonst – outer gonostylus, pm – paramere, r_1_ – first radial cell, r-m – radio-medial cross vein, rst – rostrum, tg 9 – ninth tergite, th – thorax.

**Figures 8–19.**
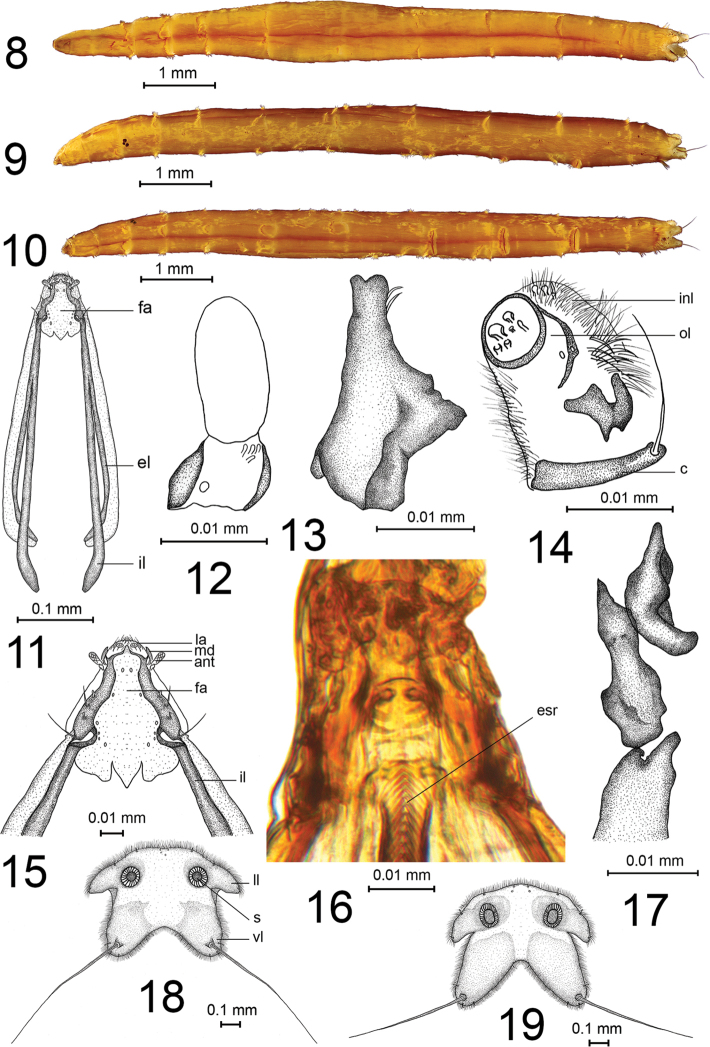
Larva of Elephantomyia (Elephantomyia). **8–18**E. (E.) hallasana Podenas, Podeniene, sp. nov., paratype **8** general view, dorsal aspect **9** general view, lateral aspect **10** general view, ventral aspect **11** head, dorsal view **12** antenna **13** mandible **14** maxilla **15** frontoclypeus **16** esophageal region **17** hypopharynx **18** spiracular field **19** spiracular field of E. (E.) edwardsi. Abbreviations: ant – antenna, c – cardo, el – externolateralia, esr – esophageal region, fa – frontoclypeal apotome, il – internolateralia, inl – inner lobe, la – labrum, ll – lateral lobe, ol – outer lobe, s – spiracle, vl – ventral lobe.

***Paratypes***: 1 male (in EtOH), 1 female (pinned), 2 larvae (one dissected and slide-mounted), South Korea, Jeju Island, Hallasan National Forest, 33°25.93'N, 126°35.87'E, alt. 580 m, 2017.05.24, S. Podenas, V. Podeniene leg. (NIBR); 1 female (pinned), South Korea, Jeju-do (do = Island), Jeju-si, Yonggang-dong, 33°25.83'N, 126°35.84'E, alt. 590 m, [at margin of Hallasan National Forest], 2017.05.24, S. Podenas, V. Podeniene leg., at light (NIBR); 3 females (pinned), 2 females (in EtOH), topotypic (NIBR).

**Figure 20.**
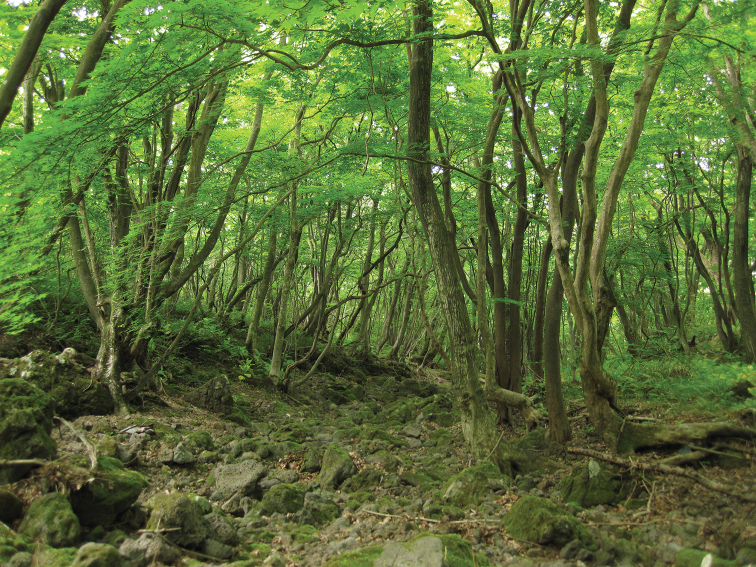
Habitat (type locality) of Elephantomyia (Elephantomyia) hallasana Podenas, Podeniene, sp. nov.

#### Comparative material examined.

**Figures 21–26.**
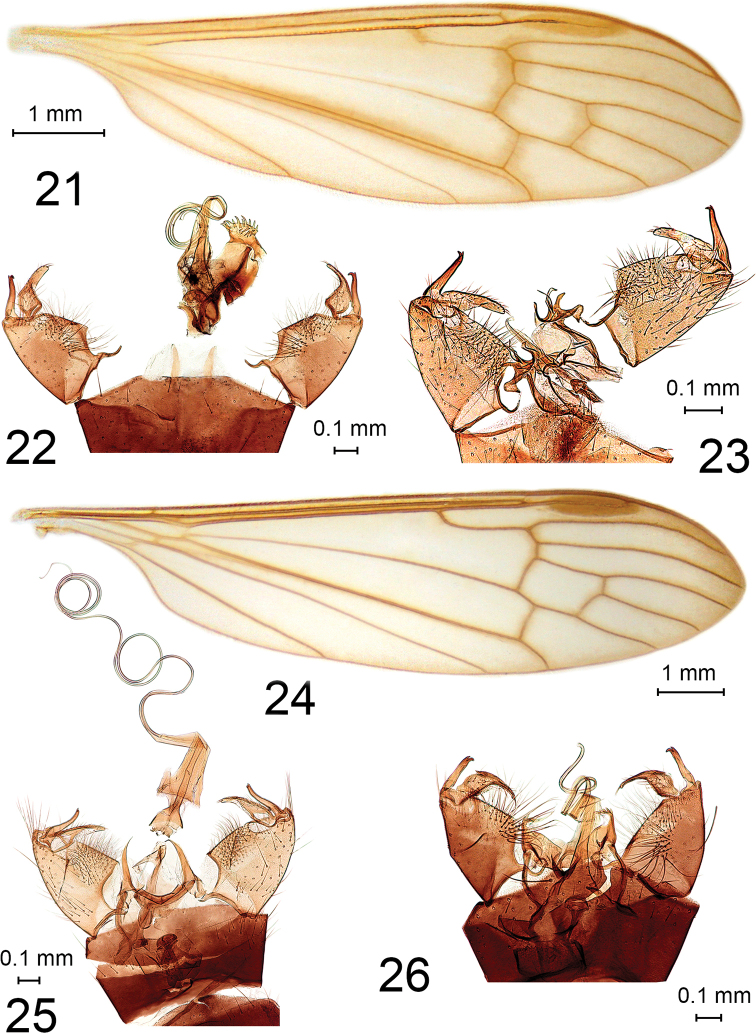
Elephantomyia (Elephantomyia). **21**E. (E.) carbo, holotype, wing **22**E. (E.) palmata, holotype, male genitalia, dorsal view **23**E. (E.) tetracantha, holotype, male genitalia, dorsal view **24**E. (E.) hokkaidensis, wing **25**E. (E.) hokkaidensis, male genitalia, dorsal view **26**E. (E.) dietziana, paratype, male genitalia, dorsal view.

*E. carbo carbo*: holotype, male (slide-mounted) (Fig. 21), China, Czechwan, Mount Omei, White Cloud Temple, alt. 2743 m, 1937.06.12, Tsen leg. (USNM); *E. dietziana*: paratype, male (slide-mounted) (Fig. 26), Japan, Kiushiu, Kirishima, alt. 762 m, 3 May 1929, S. Issiki leg. (USNM); *E. hokkaidensis*: metatype, male (slide-mounted) (Figs 24, 25), Japan, Kiushiu, Iwate, Funakosi, 20 September 1947, H. Yamamoto leg. (USNM); *E. insolita*: holotype, female (slide-mounted), China, Czechwan, Mount Omei, Chu Lao Tong Temple, alt. 1981 m, 6 June 1938, Tsen leg. (USNM); *E. palmata*: holotype, male (slide-mounted) (Fig. 22), Japan, Honshiu, Ontake, Hida, 6–10 July 1934, H. Ise leg. (USNM), paratypes: male (pinned, parts slide-mounted), Japan, Honshiu, Ontake, Hida, Southern Alps, alt. 1800 m, 10 July 1934, H. Ise leg. (USNM), male (pinned), Ontake, Hida, 6–10 July 1934, H. Ise leg. (USNM); *E. plumbea* (as *E. dietziana plumbea*): allotype, male (slide-mounted), Japan, Shikoku, Imanoyama, alt. 865 m, 12 May 1951, Issiki-Ito leg. (USNM); *E. tetracantha*: holotype, male (slide-mounted) (Fig. 23), Japan, Shikoku, Mt. Tsurugi, 31 May 1950, Issiki-Ito leg. (USNM); *E. edwardsi* (Figs 3, 7) and *E. subterminalis* (Fig. 4) listed in Podenas et al. (2015).

#### Diagnosis.

***Adult.*** It is a brown to light-brown species with banded abdomen. Body length 6.7–10.5 mm. Head gray and bearing rostrum that is approximately as long as abdomen. Mesonotal prescutum has distinct median and indistinct lateral stripes. Pleuron dark brown. Wing unpatterned except elongate light brown stigma. Abdominal tergites yellow to yellowish brown frontally, dark brown posteriorly, pattern more distinct in male. Abdomen of female darker than that of male with a very distinct light-yellow spot on the seventh tergite. Male genitalia with elongate gonocoxite, distal portion of which extends distinctly beyond bases of gonostyli. Outer gonostylus slightly angulate medially, with apex turned outwards and bearing small subapical tooth, inner gonostylus wide and non-sclerotized. The paramere armed with 4 or 5 teeth.

***Larva.*** Medium-sized, 9–17 mm long. Body covered with long, golden hairs. Head capsule reduced, weakly sclerotized, elongated, posterior part consists of two pairs of rods. Mandible small, with two prominent apical teeth; antenna long, apical segment much longer than basal. Esophageal region strengthened with oblique parallel ctenoid sclerotized structures. Spiracular disc with lateral and ventral lobes, entirely covered with pale sclerites. Ventral lobe bears long apical seta.

#### Description.

***Adult*** (Fig. 1). General body color brown to light brown. Body length of male 6.7–8.8 mm, female 7.0–10.5 mm. Male wing: 6.5–7.4 mm, female wing: 6.7–9.2 mm.

*Head.* Gray, posterior yellowish. Vertex narrow, covered with sparse golden setae. Length of male antenna 1.2–1.3 mm, female 1.1–1.5 mm. Scape dark brown dusted with gray, approximately as long as wide. Pedicel nearly rounded, light brown. Flagellum with two basal segments brownish, remainder pale yellow. Flagellum 14-segmented. Basal flagellomere large, rounded. Segments decreasing in width towards apex, apical flagellomere very small. Longest flagellomeres at the middle of antennae. Verticils up to three times as long as respective segments. Short erect pubescence, covering segments, pale. Rostrum brown, as long as abdomen, covered with short brown pubescence. Male rostrum approximately 5.0 mm, female 6.1–8.6 mm long. Palpus very short, three-segmented, basal segment longer than two succeeding segments combined. Labella pale yellow.

*Thorax.* Brown to light brown, covered with sparse brownish gray-pruinosity. Cervical sclerites dark brown. Pronotum dark brown dorsally, yellow laterally, dusted with brownish pruinosity. Mesonotal prescutum with wide brown median line, darker anteriorly and less distinct posteriorly. Lateral stripe short, indistinct. Wing (Fig. 6) translucent, with weak brownish tint, slightly yellowish at base and along entire costal field. Stigma light brown, elongate. Veins brown. Wing venation: vein Sc long, tip of Sc_1_ at branching point of radial sector, Sc_2_ close to Sc_1_ tip, Rs comparatively short, angulate and short spurred at base, R_2_ (r-r) missing, cell r_1_ widened distally; both branches of Rs slightly arched, tips bent posteriorly; cross-vein r-m short; discal cell large, nearly rectangular, twice as long as wide, basal deflection of CuA_1_ at the middle of discal cell, both anal veins nearly straight, diverging, anal lobe long, slightly widened at middle. Halter brownish yellow, pale at base. Male halter 0.9–1.1 mm long; female 1.0–1.4 mm. Femur brown with yellow base, dark brown distally, tibiae brown, indistinctly darkened at apex, tarsomeres light brown, distal segment darker. Legs covered with short, semi-adherent, brownish setae. Male femur II: 4.9 mm, tibia II: 4.2 mm, tarsus II: 3.6 mm, female femur I: 4.2–5.6 mm long, II: 5.9 mm, III: 4.4–5.8 mm, tibia I: 6.0–7.3 mm, II: 6.6 mm, III: 5.3–6.8 mm, tarsus I: 6.2–7.3 mm, II: 5.2 mm, III: 4.6–5.0 mm long. Claw simple without subbasal spines or teeth in both sexes.

*Male abdomen.* Distinctly bicolored, tergites and sternites yellow at base and distinctly dark brown along posterior margin. First tergite darkened at base and along distal margin, with yellow spot medially. Male terminalia (Fig. 2) dark brown. Sclerites of ninth segment fused and forming complete ring, posterior margin dorsally with wide and deep invagination. Dorsal surface of ninth tergite with two densely setose emarginations. Gonocoxite long and slightly arched with two pairs of gonostyli attached slightly beyond midpoint of gonocoxite. Distal part of gonocoxite, beyond bases of gonostyli, large, with rounded apex. Outer gonostylus slightly angulate medially with apex turned outwards, distal apex darkened and distinctly bidentate. Inner gonostylus longer than outer gonostylus, wide, fleshy, and setose. Paramere with four or five long spines distally forming a comb-like structure. Aedeagus shaped as a long, coiled tube.

*Female abdomen.* Generally darker than in male, somewhat glossy. Transverse yellow sutures on tergites vary depending on specimen, but narrower than in male, less distinct on basal segments and well developed on posterior segments. Sutures on tergites more distinct laterally, but narrower and less distinct along middle of sclerite. Distinct yellow lateral spots present on 3–7, 5–7, or only on seventh tergite (Fig. 1). Tergites and sternites brown basally, dark brown distally. Seventh tergite distinctly yellow with narrowly darkened posterior margin, covered with sparse, dark brown, erect setae. Seventh sternite dark brown with narrow yellow transverse suture at base. Tenth tergite dark brown basally, rusty brown distally, covered with sparse brownish pruinosity. Cercus brown, paler at base, long and narrow, distal part raised. Eighth sternite glossy dark brown, hypovalva brown, pale apex, long and narrow, reaching to about two-thirds of cercus.

***Larva.*** Body brownish yellow (Figs 8–10). Length 7.8–8.6 mm, width 0.9 mm.

*Head.* Head capsule 0.6 mm long, 0.15 mm wide, hemicephalic, elongated, weakly sclerotized and depressed dorsoventrally (Fig. 11). Genae reduced, posterior part of head capsule consists of one pair of rod-shaped internolateralia and one pair of rod-shaped externolateralia, all bent medially, internolateralia and externolateralia joined by membrane. Labrum narrow, transversal, with numerous long hairs on epipharynx and a pair of sclerotized, comb-shaped premandibles, pair of sensory rings with two sensory papillae situated on anterior part of labrum (Fig. 15). Frontoclypeal apotome membranous with a pair of sensory pits on anterior portion, a pair of similar structures anterolaterally and four pairs of pits on lateral part. Mandible slender (Fig. 13), ventral and dorsal edges without prominent teeth, two prominent apical teeth, medially with two long acute spines. Maxilla (Fig. 14) short and weakly sclerotized bearing inner (fused galea and lacinia) and outer lobes, cardo long and narrow, with a single long apical seta. Inner lobe elongate-oval, with numerous apical hairs, with large area bearing small sensory structures distally, and with elongated narrow sclerite on inner margin. Outer lobe cylindrical with apical sensory structures, with numerous hairs on apical and lateral parts and with large irregularly shaped sclerite at the base. Antenna long, reaching apex of mandible, one-segmented with four short sensory papillae and one large apical papilla. Basal segment subcylindrical, short and sclerotized, apical papilla sculptured, elongate-oval and nearly twice as long as basal segment (Fig. 12). Both antennae close to each other. Ventral side of head with numerous long hairs in the maxillary area. Hypopharynx consists of two pairs of rods (Fig. 17). Labium membranous with three pairs of sensory papillae apically. Esophageal region strengthened with oblique parallel ctenoid sclerotized structures (Fig. 16).

*Thorax.* All thoracic segments wider than long, covered with long, golden, silky hairs.

*Abdomen.* First abdominal segment wider than long. Second abdominal segment 1.5 times as long as wide. Abdominal segments II–VII almost twice as long as wide. Abdominal segments V–VII with ventral creeping welt each (Figs 9, 10). Creeping welt with brown spines, arranged into longitudinal rows. All abdominal segments covered with long, golden, silky hairs.

*Anal division.* Spiracular field surrounded by four (two lateral and two ventral) lobes (Fig. 18). Lateral lobe 1.5 times as wide as long, covered with pale sclerite surrounding spiracle, three short setae located at the outer margin of lobe. Ventral lobe as long as width at the base and entirely covered by pale sclerite. Very long seta, 2.5 times as long as lobe itself, located close to apex of lobe. One short bifurcated and two short single setae located at the apical part of each lobe. Two pairs of short setae located on the dorsal margin of spiracular field. Spiracular field fringed with short tiny setae except inner margin of lateral lobes (Fig. 18). Spiracle small, rounded, distance between spiracles more than two diameters of spiracle itself. Anal field consists of two pairs of blunt, white, fleshy anal papillae, which are retracted and hardly visible in studied specimens. Tuft of very long dense hairs located in front of anal field.

#### Etymology.

The new species is named after the locality where it was collected, Hallasan National Park, which surrounds the highest mountain in South Korea, the shield volcano Hallasan.

#### Distribution.

Currently known only from Hallasan National Park, Jeju Island, South Korea.

#### Habitats.

Valley floor covered with deciduous trees and shrubs, and moss covered rocks (Fig. 20); deciduous forest with dense cover of bamboo-grass (*Sasa quelpaertensis*); park meadow with sparsely planted deciduous trees mixed with pines. Adults are attracted to light. Larvae were found under the bark of truncated deciduous tree trunks, in sap, with fungi together with Atypophtalmus (Microlimonia) sp. and *Libnotes* sp.

#### Elevation.

Less than 600 m to 1100 m.

#### Period of activity.

Adults on wing from late May through middle of June.

## Discussion

*Elephantomyia hallasana* sp. nov. is the only *Elephantomyia* species recorded from the Jeju Island. It is closely related to *E. edwardsi* (Figs 3, 7), which is recorded from the Korean Peninsula, and *E. subterminalis* (Fig. 4), which was described from Shikoku Island, Japan. The most striking difference of *E. hallasana* sp. nov. is the huge distal portion of the gonocoxite extending beyond the base of the gonostyli (Fig. 2) and the comparatively stout gonostyli. The outer gonostylus is slightly angulate medially with its apex turned outwards, not inwards as in most other species of the genus *Elephantomyia*. The distal portion of the gonocoxite is small in *E. subterminalis*, comparatively big in *E. edwardsi*, but much larger in *E. hallasana* sp. nov. The outer gonostylus bears a small subapical tooth, which usually is hidden in dorsal view in *E. edwardsi* but distinctly visible in *E. hallasana* sp. nov. The inner gonostylus in *E. edwardsi* and *E. subterminalis* has a narrow apex but fleshy and broad in *E. hallasana* sp. nov. The paramere of *E. hallasana* sp. nov. has 4 or 5 teeth, like that in *E. subterminalis*, but bears many spines, as in *E. edwardsi*. The wing venation is similar in all three species, and only the radial sector is somewhat longer in *E. edwardsi*. The female of *E. edwardsi* has a banded pattern on the abdomen, similar to that of the male; the female of *E. subterminalis* is still undescribed. The female of *E. hallasana* sp. nov. has darker abdomen than that of male and that of both sexes of *E. edwardsi*, with a very distinct, light-yellow spot on the seventh tergite. Despite *E. hallasana* sp. nov. being currently known only from Jeju Island, we expect that related species occur in the Far East of Russia. It is likely that species identified by E. N. Savchenko as *E. subterminalis* (Savchenko 1976, 1983) from the Russian Far East (Fig. 5) represents a new species, related to *E. hallasana* sp. nov. E. N. Savchenko’s illustration of male terminalia shows structures more similar to *E. hallasana* sp. nov. than to *E. subterminalis*.

Larvae of seven species of subgenus E. (Elephantomyia) are known: E. (E.) aurantiaca Alexander, 1917, E. (E.) edwardsi, E. (E.) hokkaidensis, E. (E.) krivosheinae, E. (E.) montana Alexander, 1934, E. (E.) subterminalis, and E. (E.) westwoodi westwoodi Osten Sacken, 1869, were described to date (Alexander 1920; Bangerter 1934; Wood 1952; Krivosheina 2010; Krivosheina and Krivosheina 2011). Immature stages of other three subgenera are still unknown. Based on this material, two different larval types could be distinguished: a moss-dwelling group with a massive, almost complete head capsule and reduced spiracular lobes (Afrotropical species: E. (E.) aurantiaca), and a dead-wood-inhabiting type with a strongly reduced head capsule and four-lobed spiracular field (Afrotropical, Palaearctic, and Nearctic species: E. (E.) krivosheinae, E. (E.) edwardsi, E. (E.) subterminalis, E. (E.) hokkaidensis, E. (E.) westwoodi, E. (E.) montana). These two similar morphological groups have only the ventral creeping welts on abdominal segments V–VII and the golden yellow body coloration in common; other characters are different. Elephantomyia (E.) hallasana sp. nov. shares the characters of the second, dead-wood inhabiting type.

According to Krivosheina (2010), species-specific differences of *Elephantomyia* larvae were noticed in the sclerotization pattern of spiracular fields and in spines of creeping welts. We found that species differ also in the ratio between the length of the ventral apical hair and the base width of the ventral spiracular lobe. Head capsules of different species are similar.

The larva of E. (E.) subterminalis from the Far East of Russia, Kedrovaya Pad and Ussuri Nature Reserves was described by Krivosheina (2010), but we have doubts about the determination of that species. The larva of E. (E.) hallasana sp. nov. is similar to the larva described by Krivosheina; the only difference is the length of the ventral apical seta, which is more than 2.5 times as long as the ventral spiracular lobe in E. (E.) hallasana sp. nov. and less than twice as long as lobe in the species from the Kedrovaya Pad and Ussuri Nature Reserves.

Two species of *Elephantomyia*, E. (E.) edwardsi and E. (E.) hallasana sp. nov., occur on the Korean Peninsula. Larvae of these two species differ in characters of spiracular field, such as the sclerotization of ventral lobes, ratio of length and width of the ventral lobe, and length of ventral setae. Ventral sclerites of E. (E.) edwardsi (Fig. 19) cover a larger part of the ventral lobe than in E. (E.) hallasana sp. nov. (Fig. 18), the ventral lobe in E. (E.) hallasana sp. nov. is just slightly longer than wider at base, whereas in E. (E.) edwardsi the ventral lobe is more than 1.5 times longer than wider at base, the length of ventral apical seta is more than 2.5 times as long as the ventral spiracular lobe in E. (E.) hallasana sp. nov. and less than twice as long as the lobe itself in E. (E.) edwardsi.

## Supplementary Material

XML Treatment for Elephantomyia

XML Treatment for Elephantomyia (Elephantomyia) hallasana
